# The Effect of Human Factor H on Immunogenicity of Meningococcal Native Outer Membrane Vesicle Vaccines with Over-Expressed Factor H Binding Protein

**DOI:** 10.1371/journal.ppat.1002688

**Published:** 2012-05-10

**Authors:** Peter T. Beernink, Jutamas Shaughnessy, Rolando Pajon, Emily M. Braga, Sanjay Ram, Dan M. Granoff

**Affiliations:** 1 Center for Immunobiology and Vaccine Development, Children's Hospital Oakland Research Institute, Oakland, California, United States of America; 2 Division of Infectious Diseases and Immunology, University of Massachusetts Medical School, Worcester, Massachusetts, United States of America; Northwestern University Feinberg School of Medicine, United States of America

## Abstract

The binding of human complement inhibitors to vaccine antigens *in vivo* could diminish their immunogenicity. A meningococcal ligand for the complement down-regulator, factor H (fH), is fH-binding protein (fHbp), which is specific for human fH. Vaccines containing recombinant fHbp or native outer membrane vesicles (NOMV) from mutant strains with over-expressed fHbp are in clinical development. In a previous study in transgenic mice, the presence of human fH impaired the immunogenicity of a recombinant fHbp vaccine. In the present study, we prepared two NOMV vaccines from mutant group B strains with over-expressed wild-type fHbp or an R41S mutant fHbp with no detectable fH binding. In wild-type mice in which mouse fH did not bind to fHbp in either vaccine, the NOMV vaccine with wild-type fHbp elicited 2-fold higher serum IgG anti-fHbp titers (P = 0.001) and 4-fold higher complement-mediated bactericidal titers against a PorA-heterologous strain than the NOMV with the mutant fHbp (P = 0.003). By adsorption, the bactericidal antibodies were shown to be directed at fHbp. In transgenic mice in which human fH bound to the wild-type fHbp but not to the R41S fHbp, the NOMV vaccine with the mutant fHbp elicited 5-fold higher serum IgG anti-fHbp titers (P = 0.002), and 19-fold higher bactericidal titers than the NOMV vaccine with wild-type fHbp (P = 0.001). Thus, in mice that differed only by the presence of human fH, the respective results with the two vaccines were opposite. The enhanced bactericidal activity elicited by the mutant fHbp vaccine in the presence of human fH far outweighed the loss of immunogenicity of the mutant protein in wild-type animals. Engineering fHbp not to bind to its cognate complement inhibitor, therefore, may increase vaccine immunogenicity in humans.

## Author Summary

Vaccines containing factor H-binding protein (fHbp) are being developed for protection against bacterial meningitis and sepsis caused by meningococci. The antigen was identified from genomic sequences and only later found to bind a human complement protein, factor H (fH), but not fH from non-human species. In previous studies, native outer membrane vesicle (NOMV) vaccines from mutants with over-expressed fHbp elicited broadly protective serum antibodies in mice whose fH did not bind to fHbp in the vaccine. In this study, the authors immunized transgenic mice and showed that the presence of human fH decreased serum bactericidal antibody responses to a NOMV vaccine with fHbp that bound human fH. In contrast, a NOMV vaccine containing fHbp with a single amino acid substitution that eliminated fH binding elicited nearly twenty-fold higher protective antibody responses. Thus, a simple change in a vaccine antigen to eliminate binding to a host protein can increase immunogenicity.

## Introduction

*Neisseria meningitidis* causes sepsis and meningitis with relatively high rates of fatalities or severe permanent sequelae [1], [2]. Licensed quadrivalent polysaccharide-protein conjugate vaccines are available against four capsular groups: A, C, W135 and Y. Development of conjugate vaccines against group B strains, however, has been hampered by cross-reactivity of the group B polysaccharide with host molecules [3], [4], and safety concerns about the potential to elicit auto-reactive antibodies. Development of a vaccine against group B strains is important since these strains are responsible for about one-third of cases of meningococcal disease in the U.S. [1] and up to 90% in some European countries [5].

Several non-capsular antigen-based vaccines are being developed against group B meningococci (reviewed in [6], [7]). One of the most promising antigens is factor H-binding protein (fHbp) [8], [9]. Vaccines containing recombinant fHbp [10]–[12] or native outer membrane vesicles (NOMV) from mutant meningococcal strains with over-expressed fHbp [13], [14] are being tested in humans. After clinical testing had started, fHbp was discovered to bind complement factor H (fH) [15]. Further, binding was found to be specific for human fH [16]. Binding of a host protein to a vaccine antigen could in theory decrease immunogenicity by covering important epitopes or decreasing uptake, processing or presentation of the antigen. Also, the implications of binding a human complement protein to a vaccine antigen with respect to its effect on immunogenicity or the potential safety concern of eliciting auto-antibodies had not been considered at the time of starting the clinical trials with these vaccines.

Using transgenic mice, we recently reported that the presence of human fH impaired immunogenicity of a recombinant fHbp vaccine that bound human fH [17]. In that study we also described a mutant fHbp antigen in which substitution of arginine 41 with serine (R41S) abrogated binding of fH to the fHbp vaccine. Serum bactericidal antibody responses of human fH transgenic mice immunized with the recombinant R41S mutant fHbp vaccine were higher than those of mice immunized with the recombinant wild-type fHbp vaccine that bound fH.

In wild-type mice, NOMV vaccines from mutants with genetically attenuated endotoxin and over-expressed fHbp elicited high titers of serum anti-fHbp antibodies, which had broader (i.e. in terms of strain coverage) complement-mediated bactericidal activity than anti-fHbp antibodies elicited by recombinant fHbp vaccines [18]–[23]. Thus, NOMV vaccines from mutants with increased fHbp expression appear to be a promising approach to elicit broad protective immunity against meningococcal group B strains. An important limitation of these studies was that they were done in wild-type mice whose mouse fH did not bind to the fHbp antigens. Therefore, the studies did not assess the possible effect of human fH on vaccine immunogenicity. In the present study we used human fH transgenic mice to investigate the immunogenicity of NOMV vaccines containing wild-type fHbp, or a mutant fHbp antigen that had no detectable binding of human fH.

## Materials and Methods

### Ethics statement

Vaccine immunogenicity was evaluated in mice in strict accordance with the recommendations in the Guide for the Care and Use of Laboratory Animals of the National Institutes of Health. The protocols were approved by the Institutional Animal Care and Use Committees at Children's Hospital Oakland Research Institute and the University of Massachusetts Medical School. Blood collection was performed under anesthesia, and all efforts were made to minimize suffering. The human complement source for measuring serum bactericidal activity was serum from an adult who participated in a protocol that was approved by Institutional Review Board (IRB) of Children's Hospital Oakland Research Institute. Written informed consent was obtained from the subject.

### Construction of vaccine strains

The NOMV vaccines were prepared from mutants of group B vaccine strain H44/76 (**Table 1**). The parent strain was isolated from a patient with invasive disease during an epidemic in Norway, and was used to prepare a detergent-extracted OMV vaccine [24], [25]. The wild-type H44/76 strain naturally expresses a high level of fHbp amino acid sequence variant ID 1 as classified in the public database of fHbp variants (http://pubmlst.org/neisseria/fHbp/). To attenuate endotoxin [26], the LpxL1 gene was deleted as previously described [23], which resulted in penta-acylated instead of hexa-acylated lipo-oligosaccharide (LOS) [26], [27]. The resulting mutant strain (H44/76ΔLpxL1, **Table 1**) was then transformed with one of three plasmids: pBSΔfHbp::erm [8], pBS-erm-fHbp wild-type (WT), or pBS-erm-fHbp R41S. The pBS-erm-fHbp WT plasmid was constructed by subcloning a cassette containing a modified PorA promoter, in which the polyguanine tract between the −35 and −10 sites was replaced with the corresponding sequence from the NadA gene, followed by the fHbp ID 1 gene, downstream of the erm cassette of pBSΔfHbp::erm. The R41S substitution was introduced into WT fHbp as described previously [17]. To increase over-expression of fHbp, a second copy of the cassette containing the modified PorA promoter and the WT fHbp gene was subcloned into pFP12 using the *Sph*I and *Stu*I restriction sites as described previously [28]. The modified plasmid was used to transform the strain with WT fHbp integrated into the chromosome. Another version of this plasmid containing R41S mutant fHbp was used to insert a second copy into the strain with the R41S fHbp gene integrated into the chromosome. Characteristics of the three mutants used to prepare NOMV vaccines are summarized in **Table 1**.

**Table 1:** Characteristics of strains used in this study.

Strain	PorA VR Type^a^	fHbp ID^b^	fHbp variant group^c^	Purpose
Wild-type (WT) H44/76	7,16	1	1	Parental vaccine strain with naturally high expression of fHbp
H44/76ΔLpxL1	7,16	1	1	Mutant with attenuated endotoxin, which was used to engineer second fHbp mutants
H44/76ΔLpxL1ΔfHbp	7,16	None	None	Preparation of control NOMV that lacks fHbp
H44/76ΔLpxL1 with over-expressed WT fHbp	7,16	1	1	Preparation of NOMV with over-expressed fHbp that binds human fH
H44/76ΔLpxL1 with over-expressed R41S mutant fHbp	7,16	R41S mutant of ID 1	1	Preparation of NOMV with over-expressed mutant fHbp that does not bind human fH
H44/76 mutant with heterologous fHbp to vaccines	7,16	28	3	Control bactericidal test strain for antibodies directed at antigens other than fHbp in variant group 1
WT Cu385 with heterologous PorA VR to vaccines	19,15	1	1	Bactericidal test strain for antibodies directed at fHbp in variant group 1

a PorA variable region (VR) type as described Sacchi [43].

b fHbp ID from peptide database at http://pubmlst.org/neisseria/fHbp.

c Variant group as described by Masignani [8].

### Characterization of vaccine strains

Binding of human fH to the surface of live bacteria was determined by flow cytometric detection of fluorescence, which was performed as described previously [29]. Bacteria were grown to mid-exponential phase in Mueller-Hinton broth supplemented with 0.25% glucose and 0.02 mM cytidine monophosphate N-acetyl neuraminic acid (CMP-NANA), harvested by centrifugation, and suspended in blocking buffer (Dulbecco's PBS containing 1% (w/v) BSA) at approximately 7.5×10^8^ cfu/ml. Human fH (10 µg/ml) or, as controls, anti-fHbp mAb (4 µg/ml), or anti-PorA mAb (1 µg/ml) was incubated with the cells for 30 min at room temperature. The anti-fHbp mAb was JAR 5 [30] and the anti-PorA mAb was P1.7 (National Institute for Biological Standards and Controls, Potters Bar, UK; NIBSC code: 01/514) [31]. To detect bound human fH, the cells were incubated with affinity purified goat anti-human fH (2 µg/ml), followed by AlexaFluor 488 rabbit anti-goat IgG H+L (1∶500; Invitrogen). To detect murine mAbs, the cells were incubated with AlexaFluor 488 goat anti-mouse IgG H+L (1∶500; Invitrogen). The preparations were fixed with 0.5% formaldehyde in PBS buffer, and the bacterial cells were analyzed by flow cytometry (LSRFortessa, BD Biosciences).

### Production and characterization of NOMV vaccines

NOMV vaccines were prepared from membrane blebs released into bacterial culture supernatants as previously described [32]. To test binding of fH or mAbs by ELISA, the NOMV vaccines (2 µg/ml in PBS) were adsorbed to the wells of a microtiter plate (Immulon 2HB; Thermo Scientific) by incubation overnight at 4°C. Non-specific binding was blocked with PBS containing 1% BSA for 1 h at room temperature. Different concentrations of purified human fH (Complement Technology) were added to the plate, and were incubated for 2 h at room temperature. After washing, bound fH was detected with goat anti-human fH (affinity purified, 1 µg/ml) followed by rabbit anti-goat IgG conjugated to alkaline phosphatase (Sigma; 1∶5,000). Murine mAb binding to the NOMV vaccines was tested by a similar procedure except that the mAbs (anti-fHbp mAb JAR 5 or anti-PorA mAb P1.7) were added instead of fH, and bound mAbs were detected with goat anti-mouse IgG conjugated to alkaline phosphatase.

We characterized the three NOMV preparations further by Western blotting to detect fHbp expression, and by SDS-PAGE. The proteins in the NOMV preparations were separated by SDS-PAGE (10% acrylamide NuPAGE; Invitrogen) and stained with Coomassie blue (SimplyBlue; Invitrogen). For the Western blot, the proteins were transferred to a polyvinylidene fluoride membrane (Immobilon-FL; Millipore) using an XCell II Blot Module (Invitrogen). The membrane was blocked using PBS (Roche) containing 1% (w/v) blocking grade nonfat dry milk (Bio-Rad), and then washed with PBS containing 0.1% Tween-20 (Sigma). fHbp was detected using anti-fHbp mAb JAR 5 (0.1 µg/ml) and goat anti-mouse IgG conjugated to IRDye 800CW (Li-Cor Biosciences). The IRDye was detected at 800-nm wavelength on an infrared scanner (Odyssey; Li-Cor Biosciences).

### Immunogenicity in wild-type mice

Groups of sixteen to twenty female BALB/c mice, aged 5 weeks, were immunized with one of three NOMV vaccines (fHbp knock-out (KO); over-expressed wild-type fHbp (OE WT); or over-expressed R41S mutant fHbp (OE R41S)). As described above, all three vaccines were prepared from mutants in which the LpxL1 gene had been inactivated to attenuate endotoxin activity [27], Each NOMV vaccine dose contained 2.5 µg of protein and 600 µg of aluminum hydroxide (Alhydrogel; Brenntag Biosector). Additional groups of control mice were immunized with 20 µg of recombinant fHbp ID 1 (N = 10 mice) or aluminum hydroxide adjuvant alone (N = 7 mice). Two doses were given intraperitoneally at three-week intervals and blood was collected by cardiac puncture three weeks after the second dose.

### Immunogenicity in human fH transgenic mice

Details of the construction and characterization of human fH transgenic mice have been described [17]. In the present study, the number of available human fH transgenic BALB/c mice was limited, in part because we excluded mice with serum human fH concentrations <240 µg/ml as measured by a fHbp capture ELISA [17]. The rationale for exclusion was our previous data that suggested that the effect of human fH on decreasing fHbp immunogenicity was greatest in mice with high serum human fH concentrations (>250 µg/ml, with the typical range of fH concentrations in sera from humans being between 200 to 400 µg/ml [17]). To maximize the sizes of the treatment groups of fH transgenic mice, we included both male and female animals, ages 2 to 4 months. The 32 available mice were randomized to one of four vaccine groups using the random number function in Microsoft Excel. Thirteen animals were assigned to receive the NOMV vaccine with over-expressed WT fHbp, and 13 animals to receive the NOMV vaccine with over-expressed R41S mutant fHbp. The remaining six mice served as controls (three were immunized with aluminum hydroxide adjuvant alone and three were immunized with an NOMV vaccine from a fHbp knock-out mutant).

For the immunogenicity study in human fH transgenic mice, three doses of NOMV vaccine (2.5 µg of protein per dose) were given intraperitoneally at three-week intervals. Blood was collected by cardiac puncture three weeks after the third dose at which time the animals were euthanized. During the study, five mice were removed before completing the vaccination schedule (three in the group immunized with the NOMV vaccine with over-expressed R41S fHbp, one in the group immunized with the NOMV vaccine from the fHbp KO, and one in the group immunized with aluminum hydroxide). These animals either were males injured from fighting and were euthanized to minimize pain or distress, or died for other reasons not related to the procedures.

### Serology

Sera from individual mice were tested for IgG anti-fHbp antibody titers by ELISA, which was performed as described previously [33]. Complement-mediated serum bactericidal activity was measured using washed, exponential growth-phase bacteria grown to OD_620_ = 0.6 in Mueller-Hinton broth supplemented with 0.25% glucose and 0.02 mM CMP-NANA. The group B test strain, Cu385, expressed fHbp ID 1 that matched the amino acid sequence of fHbp ID 1 in the vaccines, and had a PorA variable region (VR) type heterologous to strain H44/76, which was used to prepare the NOMV vaccines (**Table 1**). For measuring bactericidal activity, the mouse sera were heated at 56°C for 30 min to remove endogenous complement activity. The exogenous complement source was human serum from a donor lacking intrinsic bactericidal activity. To eliminate possible effects of naturally acquired non-bactericidal IgG antibodies in the human complement on the bactericidal titers of the mouse sera [34], the human serum was depleted of IgG by passing one ml of serum over a protein G column (HiTrap Protein G HP 1 ml; GE Healthcare) as previously described [17]. The final 40-µl bactericidal reaction included different dilutions of the mouse sera, 20% (v/v) human complement and ∼10^3^ cfu of the test strain. After one hour of incubation at 37°C in the presence of 5% CO_2_, 12 µl of the reaction were plated. Titers were assigned by the interpolated dilution of test serum that gave 50% survival of the bacteria after 60 min incubation relative to CFU of negative controls at time 0.

Adsorption of serum anti-fHbp antibody was performed using recombinant fHbp, or recombinant human albumin as a mock treatment, each coupled separately to CnBr-activated Sepharose 4B (Sigma), which was performed as described [34]. Two serum pools were prepared from wild-type mice immunized with the NOMV vaccine with over-expressed WT fHbp and two serum pools from mice immunized with the NOMV vaccine with over-expressed R41S mutant fHbp (each pool contained sera from 9 to 10 mice). The pools were diluted 1∶5 and each pool was divided into three aliquots; one aliquot was incubated with the conjugated fHbp, the second with the conjugated human albumin (mock control), and the third was retained as a non-adsorbed sample. After incubation with the conjugated Sepharose overnight at 4°C, the beads were transferred to disposable columns (Bio-Rad). Three column washes were collected and concentrated to the original sample volume by ultrafiltration (Spin-X UF 6, 10K MWCO; Corning). By ELISA, the fHbp column removed >99% of the anti-fHbp antibodies (see Results).

## Results

### Characterization of mutant strains and vaccines

**Figure 1:**
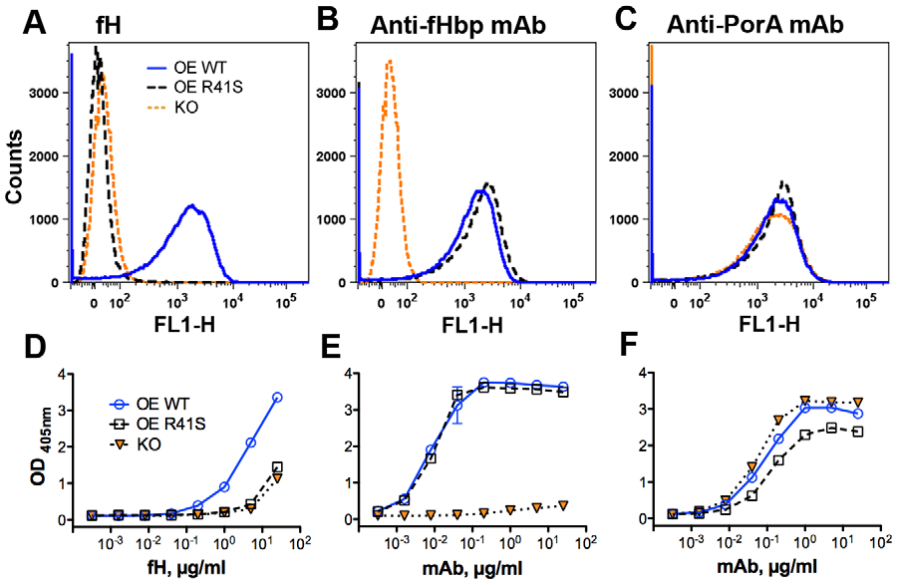
Binding of human fH or control mAbs to mutant vaccine strains and NOMV vaccines. The upper panels (**A–C**) show binding to live bacteria by indirect immunofluorescence flow cytometry. The lower panels (**D–F**) show binding to NOMV vaccines by ELISA. **A** and **D**, binding of fH; **B** and **E**, binding of anti-fHbp mAb JAR 5; **C** and **F**, binding of a control anti-PorA mAb, P1.7. WT, H44/76ΔLpxL1 bacteria or NOMV vaccine with over-expressed wild-type fHbp (blue open circles with solid blue line); R41S, H44/76ΔLpxL1 bacteria or NOMV vaccine with over-expressed R41S mutant fHbp (open squares with dashed black line); KO, H44/76ΔLpxL1ΔfHbp bacteria or corresponding NOMV vaccine with no detectable fHbp (dotted orange line (Panels A–C) or inverted orange triangles (Panels D–F)). For the flow cytometric analysis, cells with negative binding (fluorescence background values close to zero) are displayed as bi-exponential or “logical” negative values on the X axis, as described [42].

As described in the Methods, we prepared three meningococcal mutant vaccine strains for preparation of NOMV vaccines: one strain was a LpxL1 knock-out (KO) mutant with attenuated endotoxin that also had the fHbp gene inactivated (negative control); a second was an LpxL1 KO mutant engineered to over-express wild-type (WT) fHbp ID 1; and a third was an LpxL1 KO mutant with over-expressed fHbp ID 1 containing the R41S substitution to eliminate fH binding to fHbp (**Table 1**). As expected, the mutant strain with over-expressed WT fHbp bound fH (**Figure 1** **, Panel A**). In contrast, binding of fH to the mutant with over-expressed R41S fHbp was indistinguishable from that of the fHbp knock-out mutant (**Figure 1** **, Panel A**). The control anti-fHbp mAb JAR 5 showed similar binding to the strains with over-expressed WT or R41S fHbp, which showed that both of these mutants had similar levels of fHbp expression (**Figure 1** **, Panel B**). With the fHbp knock-out mutant, there was only background signal with the anti-fHbp mAb. An additional control anti-PorA mAb P1.7 bound equally well to all three strains (**Figure 1** **, Panel C**). By flow cytometry, the mutant vaccine strain with over-expressed wildtype fHbp had about 4-fold greater binding of anti-fHbp mAbs than that of the parent wild-type H44/76 strain, and 4-fold greater binding of human fH (Supplemental Figure S1, Panels A and B, respectively).

We prepared NOMV vaccines from membrane blebs released into broth culture supernatants by each of the three mutant vaccine strains using methods previously described [32]. By ELISA, there was similar binding of the control anti-fHbp mAb, JAR 5, with the NOMV vaccines from the mutants with over-expressed WT or R41S mutant fHbp, whereas there was only background binding with the NOMV vaccine from the fHbp knock-out mutant (**Figure 1** **, Panel E**). The NOMV vaccine from the mutant with WT fHbp bound ∼50 fold-more fH than the NOMV vaccine with mutant R41S fHbp (**Figure 1** **, Panel D**). Further, binding of fH to the NOMV vaccine with mutant R41S fHbp was indistinguishable from that of the NOMV vaccine from the fHbp knock-out mutant. The residual fH binding to the NOMV vaccines prepared from the mutants with over-expressed R41S fHbp or the fHbp knock-out likely was mediated by Neisserial surface protein A (NspA), which recently was identified is a second meningococcal ligand for fH [35]. The NOMV vaccines from the two mutants with over-expressed WT fHbp or fHbp knock-out had similar binding with the control anti-PorA mAb (**Figure 1** **, Panel F**). With the NOMV from the third mutant with fHbp R41S, there was slightly lower binding with the control anti-PorA mAb (**Figure 1** **, Panel F**).

**Figure 2:**
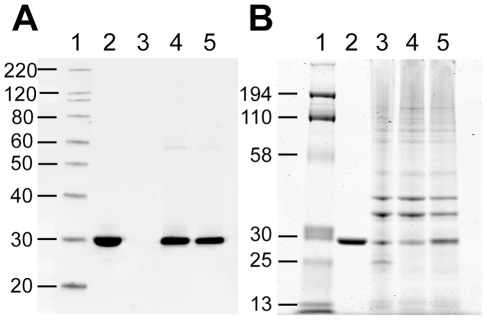
Characterization of native outer membrane vesicle (NOMV) vaccines prepared from H44/76 mutant strains. **A,** Western blot probed with an anti-fHbp mAb, JAR 5. Lane 1, molecular mass standards (Magic Mark; Invitrogen); Lane 2, purified, recombinant fHbp ID 1 (0.1 µg); Lanes 3 to 5, NOMV (0.25 µg) from H44/76 mutants. Lane 3, genetic knock-out of fHbp; Lane 4, over-expressed WT fHbp ID 1; Lane 5, over-expressed mutant R41S fHbp. **B,** SDS-polyacrylamide gel stained with Coomassie blue. Lane 1, Molecular mass standards (Kaleidoscope Prestained; Bio-Rad); lanes 2 through 5 contain the same respective samples as in Panel A except that there is 2 µg of recombinant fHbp (Lane 2) or 10 µg of NOMV (Lanes 3–5).

By Western blotting (**Figure 2** **, Panel A**), the NOMV vaccine from the fHbp knock-out strain did not contain detectable fHbp (lane 3), whereas the NOMV vaccines from the mutants with over-expressed WT or R41S fHbp contained similar amounts of fHbp (lanes 4 and 5, respectively). Although not shown in Figure 2, the amount of over-expressed fHbp in these NOMV vaccines was approximately 5-fold higher than that of an NOMV vaccine prepared in a previous study from the parent (wild-type) H44/76 strain [20]. The H44/76 strain naturally expressed relatively high amounts of fHbp [36].

By SDS-PAGE with Coomassie blue staining, the respective protein profiles of the three NOMV vaccines were similar (**Figure 2** **, Panel B**). The only exception was a band with a mass of ∼25 kDa, which was present in the NOMV from the fHbp KO mutant (lane 3) but not in the two other NOMV vaccines with over-expressed fHbp (lanes 4 and 5). This band was not identified. Note also, that a 28 kDa protein co-migrated with fHbp since SDS-PAGE the 28 kDa band was present in the of the NOMV vaccine from the fHbp knock-out mutant (**Panel B, lane 3**) but not detected by Western blot (**Panel A, lane 3**). In previous experiments, a band resolving in this portion of SDS-PAGE of NOMV preparations from wild-type and mutant strains of H44/76 with over-expressed fHbp was identified by mass spectrometry to contain both fHbp and OpcA [20].

### Serum antibody responses of wild-type mice

We immunized wild-type BALB/c mice with two doses of each of the NOMV vaccines. Control mice received two doses of the recombinant fHbp ID 1 vaccine adsorbed with aluminum hydroxide, or aluminum adjuvant alone. Since mouse fH does not bind to fHbp [16], the antibody responses provided information on vaccine immunogenicity in an animal model in which mouse fH did not bind to fHbp in any of the vaccines. Although three doses of the vaccines likely would have been more immunogenic than two doses, we chose to use two doses as a more sensitive indicator of possible loss of fHbp immunogenicity from the amino acid substitution introduced to eliminate fH binding.

**Figure 3:**
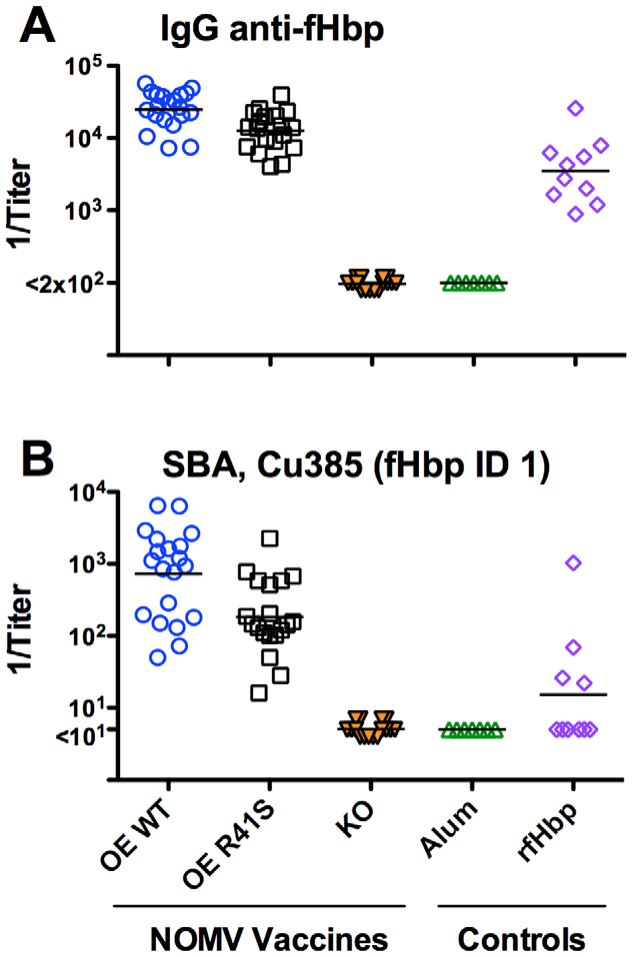
Serum antibody responses of wild-type mice immunized with NOMV vaccines. Each symbol represents the reciprocal serum titer of an individual mouse, and the horizontal line represents the geometric mean titer. OE WT, OE R41S or KO, NOMV vaccines with over-expressed wild-type fHbp, R41S mutant fHbp, or fHbp knock-out, respectively. Alum, aluminum hydroxide adjuvant only. rfHbp, recombinant fHbp. **A,** Serum IgG anti-fHbp antibody responses measured by ELISA (1/GMT of OE WT vs. OE R41S, P = 0.001; OE WT or OE R41S vs. rfHbp, P≤0.0002). **B,** Serum bactericidal antibody responses against strain Cu385, which has a mismatched PorA type compared to the vaccine strains and expresses fHbp ID 1 that matches the vaccine fHbp antigen (1/GMT of OE WT vs. OE R41S, P = 0.003).

Both of the NOMV vaccines with over-expressed WT or mutant R41S fHbp elicited higher serum IgG anti-fHbp titers than mice immunized with the control recombinant WT fHbp vaccine (P≤0.0002, **Figure 3** **, Panel A**). However, the mice immunized with the NOMV vaccine with mutant fHbp had a 2-fold lower IgG anti-fHbp geometric mean titer (GMT) than the mice immunized with the NOMV vaccine with WT fHbp (P = 0.001, **Figure 3** **, Panel A**).

The NOMV vaccines with over-expressed WT or mutant R41S fHbp elicited higher serum bactericidal responses than to the recombinant fHbp vaccine. The low responses to the recombinant protein may have reflected the use of a suboptimal two-dose schedule. The mice immunized with the NOMV vaccine with over-expressed mutant fHbp had a 4-fold lower bactericidal GMT than the mice immunized with the NOMV vaccine with over-expressed WT fHbp (P = 0.003, **Figure 3** **, Panel B**). Serum bactericidal activity was not detectable in mice immunized with the NOMV vaccine prepared from the fHbp knock-out mutant (titers <1∶10). The Cu385 strain used to measure bactericidal activity had an identical fHbp sequence but a different PorA VR type than the strains used to prepare the NOMV vaccines (Table 1). Thus, the high bactericidal responses elicited by the NOMV vaccines containing fHbp suggested that the target antigen was fHbp.

**Figure 4:**
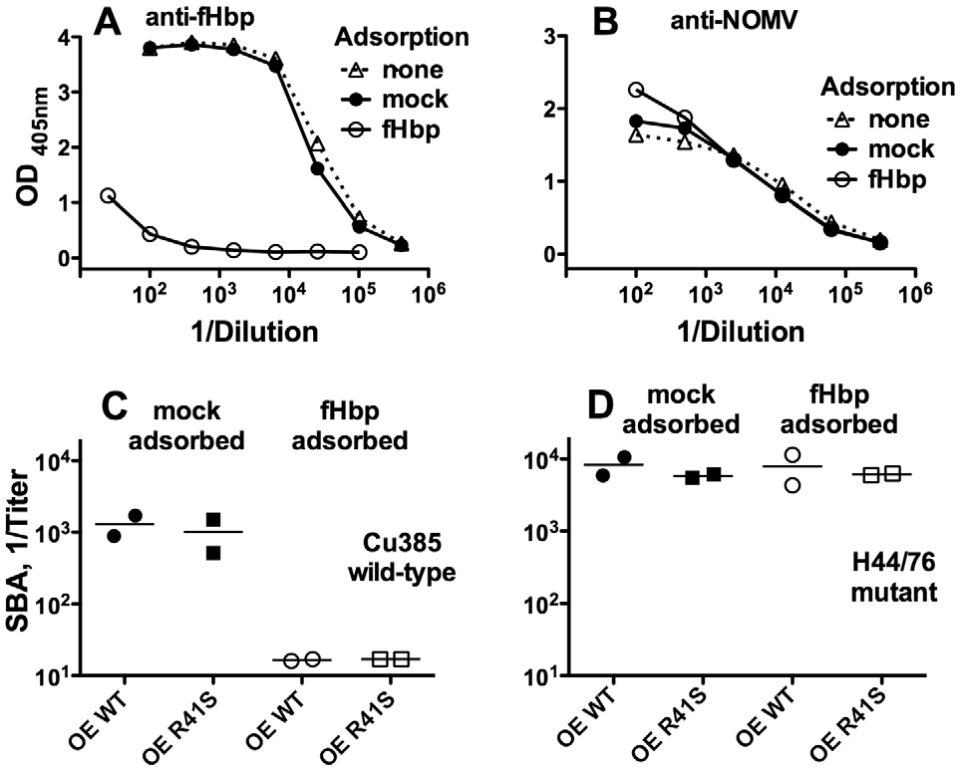
Effect of depletion of serum anti-fHbp antibodies on bactericidal activity. **A,** IgG anti-fHbp antibody as measured by ELISA. Representative data are from one of the two serum pools from wild-type mice immunized with the NOMV with over-expressed WT fHbp. The serum pool either was not adsorbed (“none,” open triangles with dotted line), or adsorbed on a human albumin column (“mock,” filled circles with solid line), or on an fHbp column (“fHbp,” open circles with solid line). The adsorbed serum samples were adjusted to the original volume applied to the column. **B,** IgG anti-NOMV antibody measured by ELISA with the NOMV vaccine prepared from fHbp knock-out strain as the antigen in the wells. Data are from the same serum pool as shown in Panel A. For panels A and B, similar respective data were obtained with each of the other three serum pools. **C,** Serum bactericidal antibody responses against strain Cu385, which has a fHbp variant group 1 antigen matched to that of the vaccines and a mismatched PorA (see Table 1). Two serum pools for each vaccine group were assayed (NOMV with over-expressed WT fHbp (“OE WT”) or R41S mutant fHbp (“OE R41S”) following depletion on mock or fHbp columns. **D.** Serum bactericidal antibody titers measured against an isogenic mutant of strain H44/76 with matched PorA to that of the vaccine strains and a mismatched fHbp in variant group 3. The respective serum pools were the same as in Panel C.

To determine the contribution of anti-fHbp antibodies to serum bactericidal activity, we depleted anti-fHbp antibodies in serum pools from mice vaccinated with the NOMV vaccines with over-expressed WT or R41S mutant fHbp by adsorption to immobilized recombinant WT fHbp (see Methods). By ELISA, the fHbp column removed greater than 99% of the anti-fHbp antibodies, compared with a negative control (mock) column containing immobilized human albumin (**Figure 4** **, Panel A**). The fHbp adsorption procedure did not affect significantly the anti-NOMV ELISA titer using NOMV from the fHbp KO mutant as the antigen adsorbed to the wells (**Figure 4** **, Panel B**). Depletion of the serum anti-fHbp antibodies resulted in a nearly 100-fold decrease in bactericidal titers against strain Cu385 with fHbp from variant group 1 (**Figure 4** **, Panel C**). As a control, we tested bactericidal activity against a second strain, which was susceptible to anti-PorA bactericidal activity (a mutant of group B strain H44/76 in which fHbp ID 28 in variant group 3 had been substituted for fHbp ID 1 in variant group 1; **Table 1**) [37]. The adsorption of the serum anti-fHbp antibody had no effect on the titers against this second strain (**Figure 4** **, Panel D**). Thus, the serum bactericidal antibodies against strain Cu385 were directed largely against fHbp.

Collectively, the immunogenicity results from the studies in wild-type mice showed that the NOMV vaccine with over-expressed mutant fHbp elicited lower serum anti-fHbp responses than the NOMV vaccine with over-expressed WT fHbp. Thus, in wild-type mice whose mouse fH did not bind to fHbp in either NOMV vaccine, the R41S substitution diminished fHbp immunogenicity.

### Serum antibody responses of human fH transgenic mice

**Figure 5:**
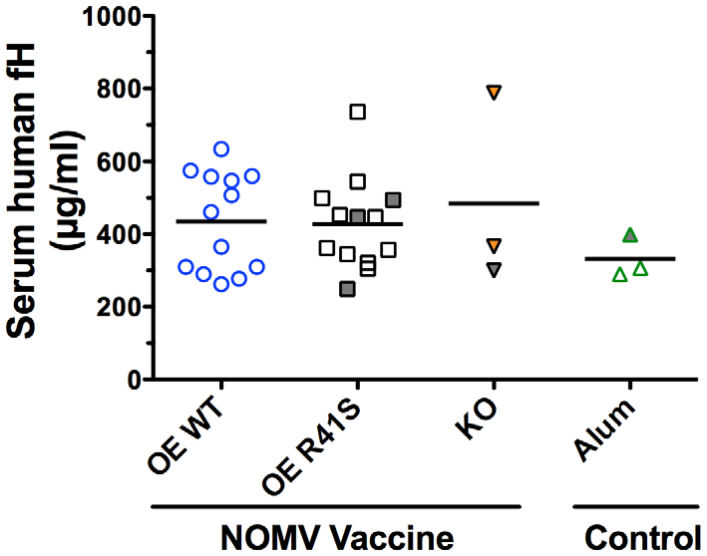
Concentrations of human fH in serum samples obtained before immunization of transgenic mice with NOMV vaccines. OE WT, mice assigned to group immunized with NOMV with over-expressed wild-type fHbp; OE R41S, mice assigned to group immunized with NOMV with over-expressed mutant R41S fHbp; KO, control mice that were immunized with NOMV from the fHbp knock-out; Alum, control mice that were immunized with aluminum hydroxide adjuvant only. Human fH concentrations of mice that were removed from the study before completion of vaccination are represented as gray filled symbols.

In humans, fH would be expected to bind to the WT fHbp vaccine antigen. To investigate the effect of human fH, we measured the immunogenicity of the NOMV vaccines in human fH transgenic mice. The respective ranges of the serum human fH concentrations of the mice in the different vaccine groups, which were measured in sera obtained before immunization, were similar (**Figure 5**), as were the respective mean ages and gender distributions of the groups.

**Figure 6:**
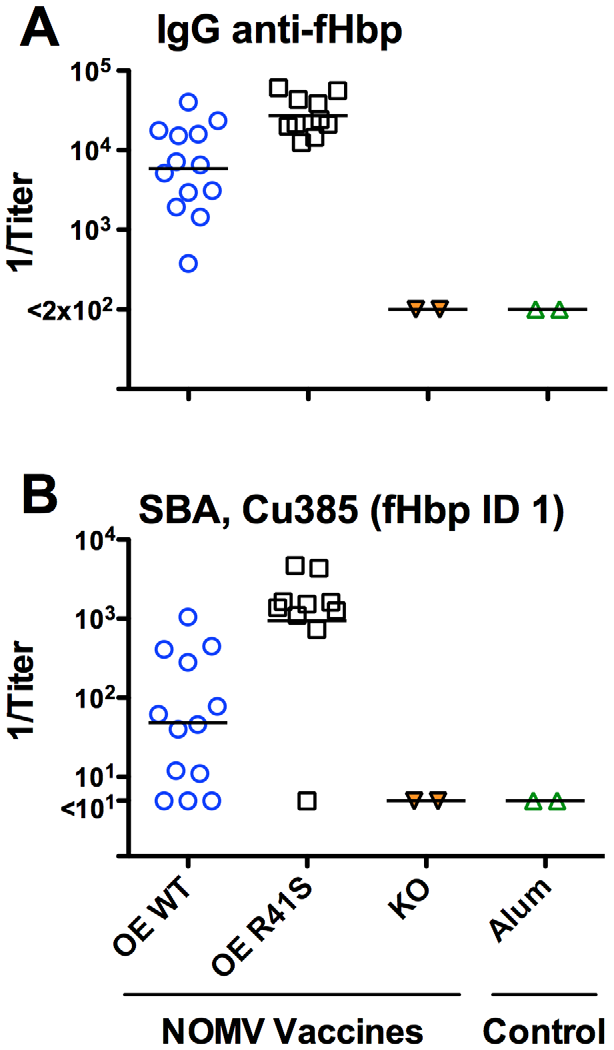
Serum antibody responses of human fH transgenic mice immunized with NOMV vaccines. Each symbol represents the reciprocal serum titer of an individual mouse, and the horizontal line represents the geometric mean titer. **A,** Serum IgG anti-fHbp antibody responses measured by ELISA. Comparing 1/GMT of OE R41S vs. OE WT, P = 0.002. **B,** Serum bactericidal antibody responses against strain Cu385 with fHbp from variant group 1. Comparing 1/GMT of OE R41S vs. OE WT, P = 0.001.

We immunized the transgenic mice with three doses of each of the NOMV vaccines. The group immunized with the NOMV vaccine with over-expressed mutant R41S fHbp had a 5-fold higher serum IgG anti-fHbp GMT (P = 0.002), and a 19-fold higher serum bactericidal GMT (P = 0.001), than the group immunized with the NOMV vaccine with over-expressed WT fHbp (**Figure 6** **, Panels A and B, respectively**). There was no correlation between the magnitude of the serum concentrations of human fH in the individual animals and the serum bactericidal titers (r = 0.15, P = 0.62). Thus, in human fH transgenic mice, there was superior immunogenicity of the NOMV vaccine with the R41S mutant fHbp, which was opposite to the results obtained in the wild-type mice.

## Discussion

In previous studies in wild-type mice, NOMV vaccines prepared from mutant meningococcal strains with attenuated endotoxin and over-expressed fHbp elicited broader serum bactericidal antibody responses than recombinant fHbp vaccines [18], [20], [21], [23]. The higher activity was particularly evident against strains with lower fHbp expression [18]. Although there are conflicting data [38], by adsorption most of the serum bactericidal antibody elicited by the NOMV vaccines against test strains with heterologous PorA VR sequence types was directed at fHbp [20], [21], [23], [28]. The underlying mechanism for the broader anti-fHbp bactericidal activity is unknown although one study suggested that the IgG anti-fHbp antibodies elicited by the NOMV vaccine had greater ability to inhibit binding of fH to fHbp than antibodies elicited by a recombinant fHbp vaccine [18]. These results suggested that the serum anti-fHbp antibody repertoire to the NOMV vaccine better targeted the fH-binding surface, as compared with other sites on the fHbp molecule when recombinant fHbp vaccines were used [18].

In the present study, we investigated the immunogenicity of NOMV vaccines prepared from mutants with over-expressed WT fHbp that bound human fH, or over-expressed R41S mutant fHbp that did not bind human fH. Introduction of the R41S mutation resulted in decreased fHbp immunogenicity in wild-type mice whose mouse fH did not bind to fHbp (**Figure 3**). In contrast, the NOMV vaccine with the mutant fHbp, which did not bind human fH, elicited nearly 20-fold higher serum bactericidal antibody responses than the control NOMV with wild-type fHbp that bound fH (**Figure 6**). Thus, the modest loss of fHbp immunogenicity, which was evident in the WT mice whose mouse fH did not bind to either vaccine was more than compensated for in the transgenic mice by the larger effect of human fH on decreasing immunogenicity of the NOMV vaccine containing fHbp that bound human fH. These results extend our earlier findings in human fH transgenic mice, which demonstrated an adverse effect of human fH on immunogenicity of a recombinant fHbp vaccine that bound human fH [17]. In the present study we found an even larger effect of human fH on lowering immunogenicity of the NOMV vaccine with the wild-type fHbp but, in contrast to our earlier study [17], we did not find a significant correlation between immunogenicity and serum human fH levels. The greater effect of human fH on lowering immunogenicity in the present study, and the lack of an inverse correlation with the serum human fH levels, may be explained by our exclusion of transgenic mice with low serum concentrations of human fH (<240 µg/ml), which was not done in our previous study.

Two lines of evidence indicated that the serum bactericidal activity measured in the present study against strain Cu385 was directed against fHbp. First, this strain was resistant to serum bactericidal antibodies elicited by the control NOMV vaccine from the fHbp knock-out strain (**Figure 3** **, Panel B**). Second, depletion of anti-fHbp antibodies from serum pools from wild-type mice immunized with NOMV vaccines that contained WT or R41S mutant fHbp resulted in ≥98% loss of bactericidal activity (**Figure 4** **, Panel C**). Similar adsorption studies were not feasible with the sera from the human fH transgenic mice because of insufficient volumes from the fewer animals in each vaccine group than used in the studies of the WT mice. We expect, however, that the serum bactericidal responses of the transgenic mice against strain Cu385 also depended on the presence of antibodies to fHbp.

The recombinant R41S mutant fHbp vaccine used in our previous study was slightly less immunogenic in wild-type mice than the recombinant WT fHbp vaccine, although the difference was not statistically significant [17]. In the present study, we immunized larger groups of wild-type mice and detected statistically significant 2-fold lower IgG anti-fHbp titers and 4-fold lower bactericidal titers elicited by the NOMV vaccine with over-expressed R41S mutant fHbp, compared with NOMV vaccine with WT fHbp. Thus, substitution of serine for arginine at residue 41 in fHbp ID 1, which resulted in no detectable binding of human fH, had an adverse effect on fHbp immunogenicity in mice lacking human fH. Potentially, alternative mutant fHbp molecules can be identified that eliminate fH binding without this loss of immunogenicity and that these vaccines will be even more effective than the R41S mutant fHbp vaccine.

Vaccines containing fHbp molecules that bind human fH are reported to elicit serum bactericidal antibodies in infants and adults and, therefore, are likely to confer protection against disease [10], [11]. Next-generation, recombinant chimeric fHbp molecules, which combine domains or regions from different variants, offer the prospect of extending protection against strains with fHbp from different variant groups [39], [40]. NOMV vaccines from mutant strains with over-expressed fHbp also can be used to increase breadth of protection [19], [21]. The present and previously published data from studies in human fH transgenic mice [17] indicate that the immunogenicity of fHbp antigens in humans also can be improved by incorporating mutations that decrease or eliminate fH binding.

Finally, a number of other pathogenic microbes including *N. gonorrhoeae, Borrelia burgdorferi, Francisella tularensis, Haemophilus influenzae, Streptococcus pneumoniae, S. pyogenes and Yersinia enterocolitica* are reported to bind complement factor H to their surface (Reviewed in [41]). Although the amino acid sequences of the fH ligands of these pathogens are not homologous to fHbp, genetic approaches similar to those employed in the present study could be employed to eliminate fH binding of these antigens and potentially increase their immunogenicity in humans. The results of testiing fHbp immunogenicity in human fH transgenic mice highlight the potential value of this mouse model for testing immunogenicity of these vaccines.

## Supporting Information

Figure S1 **Binding of anti-fHbp mAb and human fH to live bacteria of** ***N. meningitidis*** **strains as measured by flow cytometry.** Panel A. Binding of anti-fHbp mAbs (JAR 4 and JAR 5; 2 µg/ml of each). Panel B, Binding of human fH (2 µg/ml). Symbols for H44/76 strains: Wild-type strain (solid red line), which naturally expresses high amounts of fHbp; LpxL1 knockout mutant vaccine strain with over-expressed wild-type fHbp (blue line); LpxL1 knockout mutant vaccine strain with fHbp knocked-out (solid green). In this experiment the LpxL1 knockout mutant vaccine strain with over-expressed R41S mutant fHbp was not tested (For results with this strain, see Figure 1).
(TIF)
