# Supplementary figures and images for: The Effect of Human Factor H on Immunogenicity of Meningococcal Native Outer Membrane Vesicle Vaccines with Over-Expressed Factor H Binding Protein
Source: PLoS Pathog. 2012 May 10;8(5):e1002688. doi: 10.1371/journal.ppat.1002688 (PMC3349754; doi:10.1371/journal.ppat.1002688)

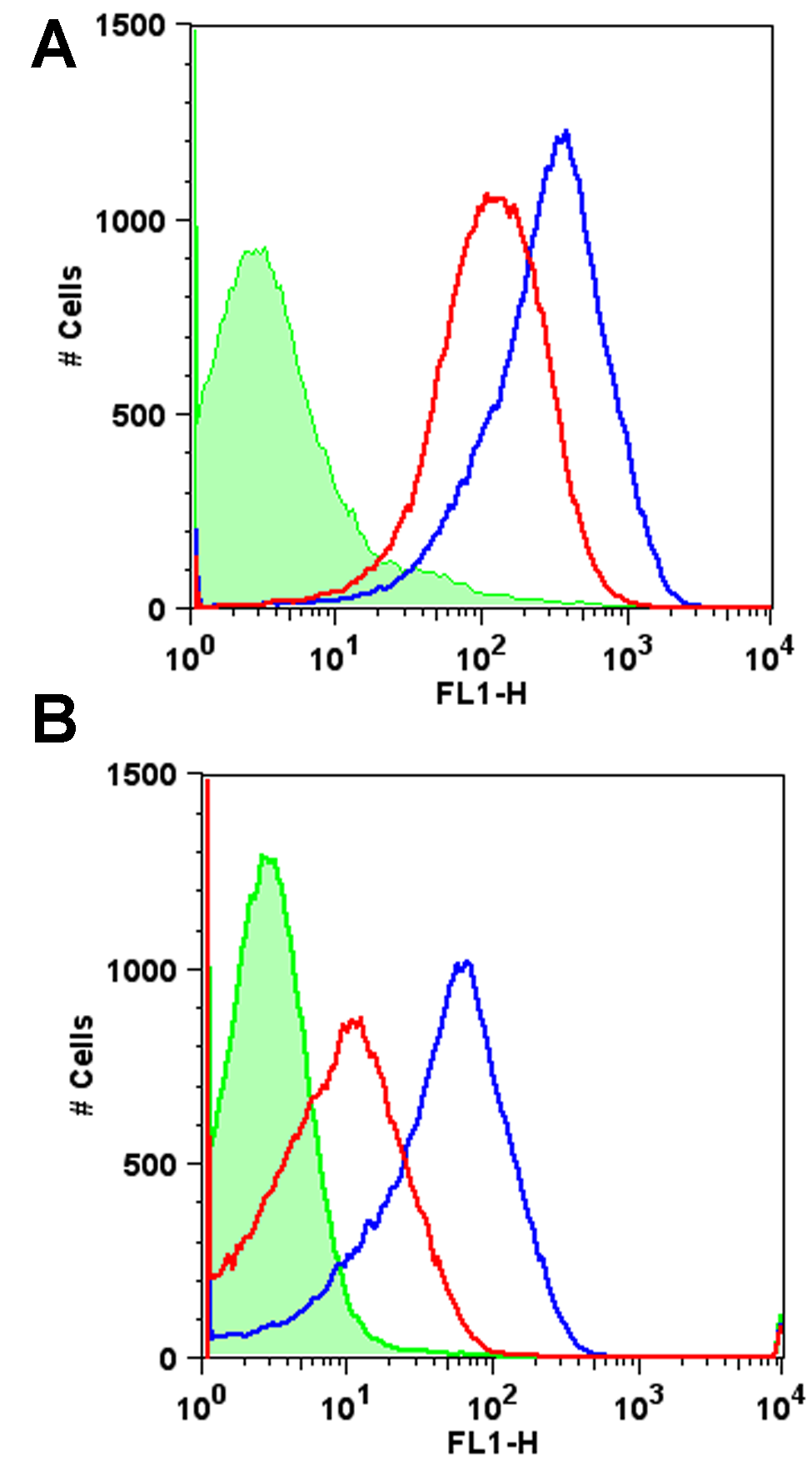

Supplement: Figure S1 — Binding of anti-fHbp mAb and human fH to live bacteria of N. meningitidis strains as measured by flow cytometry. Panel A. Binding of anti-fHbp mAbs (JAR 4 and JAR 5; 2 µg/ml of each). Panel B, Binding of human fH (2 µg/ml). Symbols for H44/76 strains: Wild-type strain (solid red line), which naturally expresses high amounts of fHbp; LpxL1 knockout mutant vaccine strain with over-expressed wild-type fHbp (blue line); LpxL1 knockout mutant vaccine strain with fHbp knocked-out (solid green). In this experiment the LpxL1 knockout mutant vaccine strain with over-expressed R41S mutant fHbp was not tested (For results with this strain, see Figure 1). (TIF) [file ppat.1002688.s001.tif]
